# Lost in Translation: The Gap in Scientific Advancements and Clinical Application

**DOI:** 10.3389/fbioe.2016.00043

**Published:** 2016-06-03

**Authors:** Joseph S. Fernandez-Moure

**Affiliations:** ^1^Department of Surgery, Houston Methodist Hospital, Houston, TX, USA; ^2^Surgical Advanced Technologies Laboratory, Department of Regenerative and Biomimetic Medicine, Houston Methodist Research Institute, Houston, TX, USA

**Keywords:** translational research, bench to bedside, clinician-scientist, FDA, NCATS, translational medicine

## Abstract

The evolution of medicine and medical technology hinges on the successful translation of basic science research from the bench to clinical implementation at the bedside. Out of the increasing need to facilitate the transfer of scientific knowledge to patients, translational research has emerged. Significant leaps in improving global health, such as antibiotics, vaccinations, and cancer therapies, have all seen successes under this paradigm, yet today, it has become increasingly difficult to realize this ideal scenario. As hospital revenue demand increases, and financial support declines, clinician-protected research time has been limited. Researchers, likewise, have been forced to abandon time- and resource-consuming translational research to focus on publication-generating work to maintain funding and professional advancement. Compared to the surge in scientific innovation and new fields of science, realization of transformational scientific findings in device development and materials sciences has significantly lagged behind. Herein, we describe: how the current scientific paradigm struggles in the new health-care landscape; the obstacles met by translational researchers; and solutions, both public and private, to overcoming those obstacles. We must rethink the old dogma of academia and reinvent the traditional pathways of research in order to truly impact the health-care arena and ultimately those that matter most: the patient.

## Introduction

The “Knowledge Doubling Curve,” coined by Buckminster Fuller, described that, by the end of World War II, the totality of human knowledge would double every 25 years (Fuller, 1982). As time has passed, this theory has become not only more evident but also more complex, with new scientific disciplines being created, each expanding at different rates. Medical knowledge, now, has a doubling time every 18 years, while newer disciplines, such as nanotechnology, double on the average of every 2 years (Densen, 2011). While the breath of knowledge in each subject varies greatly, these figures highlight the speed with which advancement now occurs.

The bench to bedside process is founded on the principle of translating findings in basic science into therapeutic interventions for patients. This “process” has been fundamental to the implementation of remarkable achievements, such as statins for dyslipidemia, targeted cancer therapy, and anti-hepatic medicines effective in HIV (Muss, 2006; Mora et al., 2010). Yet so many more bench-side success stories have found themselves stranded on the road to translation with negligible impact in the clinic. Bioengineering and biotechnology have seen a tremendous explosion of knowledge and innovation (Zucker and Darby, 1996), although these discoveries rarely materialize into FDA-approved devices or more rarely become commonly adopted by the medical community (Bagchi-Sen, 2007). The diversity of devices suffers as companies continue to recycle “old” materials to streamline the FDA approval process and meet their financial goals. New materials continue to be developed and vetted, yet the clinical impact is not felt as many clinicians rely on a handful of devices for tissue engineering and reconstruction.

Abdominal wall and orthopedic reconstruction are two of the most common surgical genres today. Over 350,000 hernia repairs are performed annually, and in 2011, ~25% of all operating room procedures performed were musculoskeletal procedures (Poulose et al., 2012; Weiss and Elixhauser, 2014). Polypropylene was invented over 60 years ago and still remains the most commonly used material in abdominal wall repair, although in use since the 1950s (Usher et al., 1958). Another example in the delay to market of bench-side materials’ success is the use of biologic or functionalized biomaterials. Regenerative medicine has reached new heights of innovation with the addition of dynamic manufacturing processes and novel drug delivery capabilities (Dimmeler et al., 2014). At the bedside, though, the use of simple acellular matrices prevails in clinical implementation, and use of functionalized biomaterials is rare (Mariette et al., 2014; Majumder et al., 2015). The unavailability, lack of options, and prohibitive costs of biologic devices has made their implementation as standard of care impossible, although they have characteristics known to be extremely favorable for tissue repair (Cevasco and Itani, 2012). Similarly, the translation of functionalized devices, often categorized as drug–device combinations, through the tireless FDA-regulatory pathways, halts their progress and ultimately their clinical implementation (Meslin et al., 2013). As we move into an era where scientific discovery and advancement occur more rapidly every year, the question lingers: why aren’t the advances at the bedside commensurate with the advances on the bench?

## Are We Speaking the Same Language?

**Figure 1:**
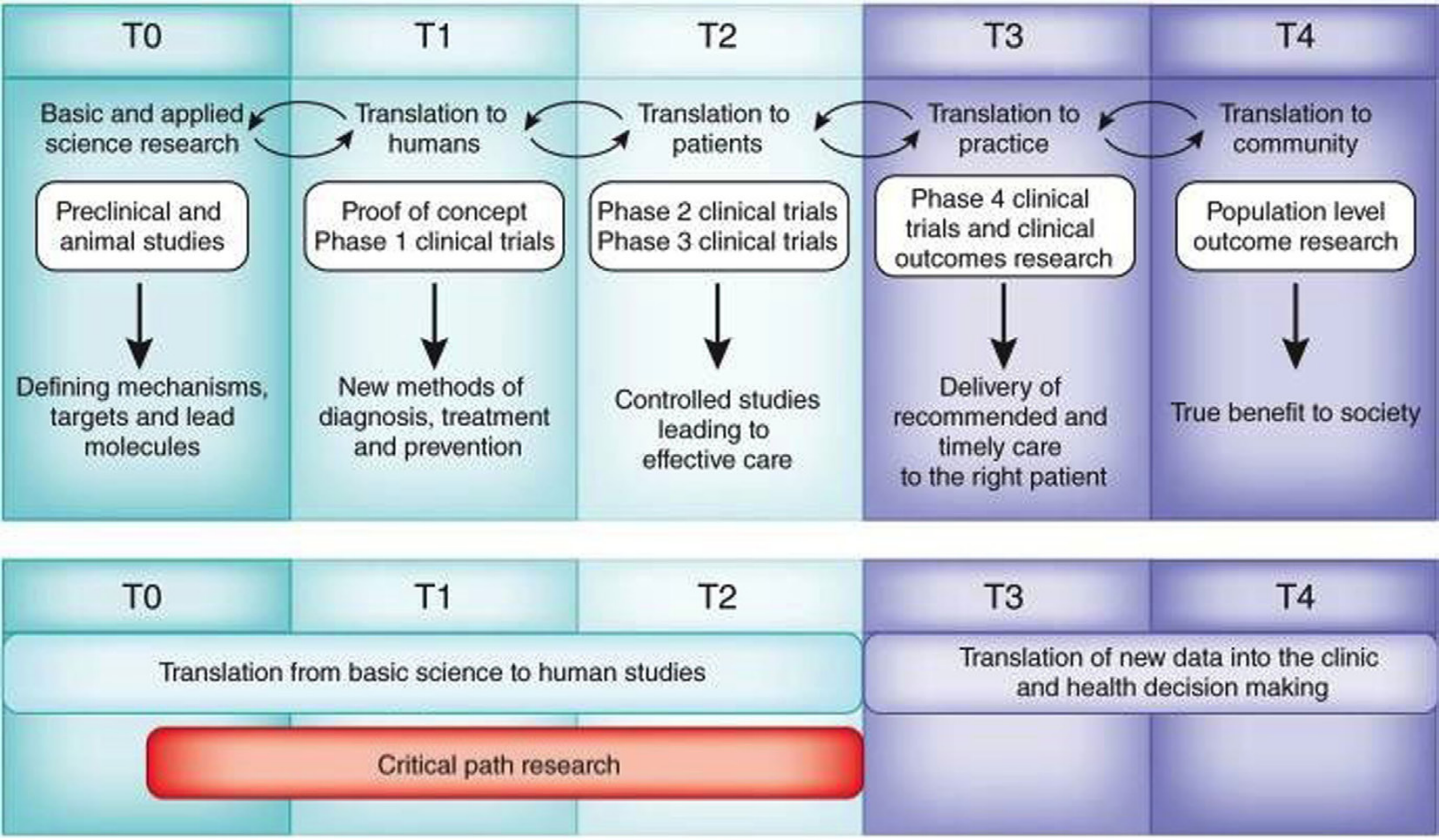
**Operational challenges for translational research and medicine**. Adapted from Blumberg et al. Harvard Catalyst Website (https://catalyst.harvard.edu/). Translational research is an iterative, dynamic, and layered process with several obstacles that must be negotiated (top). These layers include T0, identification of clinical problem followed by preclinical and optimization studies to define material candidates for compound synthesis or cellular mechanisms for intervention; T1, initial Phase I studies in humans that aim to demonstrate proof of concept and safety; T2, Phase II and III clinical trials that allowed for incremental and sequential evaluations and approvals prior to implementation; T3, post-marketing surveillance trials, conducted after the device has been in the market, are used to determine long term efficacy, impact on quality of life, and comparison to other similar devices; and T4, outcomes research to determine the impact of effectiveness intervening on patients in the general community, cost-effectiveness compared to equivalent technologies. Valley of death (red) comprises research related to the T0, T1, and T2 stages (Blumberg et al., 2012).

“Bench to bedside” has more recently evolved into the relatively new area of investigation known as *translational research*. Translational research describes the iterative process of not only basic science discoveries being integrated into clinical applications but also clinical needs and observations driving the focus of basic science (Figure 1). Fundamental to the translation of scientific discoveries to clinical impact is the collaboration and integration of scientists with clinicians, as well as the integration of academia, health care, and industry. Herein lies the problem. The cultural and academic identities of both clinicians and basic scientists are different.

Over time, two cultures have evolved in the translational landscape: the preclinical and clinical researcher. In his influential 1959 Rede lecture, British scientist and novelist, Snow, described “The Two Cultures.” The thesis of this lecture was that “the intellectual life of the whole of western society” was split into two cultures: the humanities and the sciences (Snow, 2012). While exaggerated, it draws attention to how intelligent people can differ greatly based on their perspective and backgrounds. Similarly, the culture of preclinical work and clinical work differs. Most basic scientists rarely step foot in hospitals while few physicians carry out any “wet” lab work past undergraduate or medical school, well before they have gained a true understanding of clinical needs. How hypotheses are generated, how they are tested, and how they are abandoned vary greatly between the two. One example of this lies in the current “bench to bedside” paradigm. Phase I clinical trials are the initial testing ground for novel treatments that have been found successful and potentially profitable. They provide great opportunity for groundbreaking bench work to truly prove useful, yet few rarely materialize as Phase II studies due to minimal clinical benefit demonstrated and loss of enthusiasm as the next wave of bench to bedside therapeutics hits the clinical shore.

Another example of disconnection occurs at the Phase III level. In clinical research, if one of the two blinded randomized trials fails to show benefit or either fails the entire study, testing is abandoned. This concept of halting a study based on a final pivotal experiment is absent from preclinical work where it would be unheard of to stop an entire line of study because of one result. Again, we see where the current scientific environment fails us. In this scenario, one would be inclined to learn why Phase II or III failed and study the root cause of less than expected efficacy, yet there is no room, money, or time to pursue why things did not work as planned. Further, because the scientific literary community is not interested in publishing negative results, many of the finding never make it to paper to instruct and guide those on the next wave of trials.

## Lifting the Silos

The success of any translational research program lies in the elimination of silos segregating scientists, doctors, and industry professionals from each other. While this seems intuitive to those engaged in translational medicine, the reality is that in most nations, revenue streams are strictly separated between the “hospital” and “research institute.” As the landscape of health care and reimbursement continues to evolve, clinicians will continue to be seen as earners with little to no incentive to spend any additional time pursuing innovative collaborative relationships in science. This has lead to a deficiency in the development of clinician-scientists and translational science collaboration.

The US has been a leader in the implementation of translational research partnerships for over 100 years. Since the Flexner Report was released, 105 years ago, the tradition of integrating university-owned hospitals in the research and teaching process has been a pillar to medical education and scientific advancement (Beck, 2004). As a model for this paradigm, Johns Hopkins exemplified the system of medical education where university hospitals were coupled to research-oriented schools and scientific institutes. This model is seen across the nation in such academic institutions as Harvard, Stanford, and Johns Hopkins, to name a few. Unfortunately a new breed of medical schools has emerged with a distinct separation between academia and health care, which is challenging the long-trusted academic paradigm. Frequently, clinicians are challenged to decide between “academic” and “private” clinical practice. This further widens the divide between the clinician and the scientist, a relationship crucial to success of translational medicine.

As the world embraces the need for more integrated relationships in health care and science, several initiatives have emerged to encourage partnerships. The National Institutes of Health (NIH) has led this charge with the development of the National Center for Translational Sciences (NCATS). With a focus on enabling and encouraging collaborative partnership between, not only, clinicians and scientists, but also, academia, health care, and pharmaceutical industries, NCAT focuses on bringing scientific innovation to the patient community. In 2016, the budget for Clinical and Translational Science Awards (CTSA) was $685.417 million (http://www.ncats.nih.gov/about/center/budget), indicating a substantial commitment toward the translational initiative. The international scientific community has also taken on this initiative. Germany has succeeded in promoting translational process with the Fraunhofer Institutes and combining training programs with industry partnerships. The Fraunhofer Institutes, one of the largest organizations for applied research in Europe, are strongly linked to industry with a focus on generating products for market. Similarly, in Germany, the Helmholtz Society and the Translational Centre for Regenerative Medicine (TRM) in Leipzig are designed to combine science and technology to translate science to health. Lifting the silos and promoting multidisciplinary, inter-institutional, and entrepreneurial collaboration will prove to overcome some of the current limitations, but a fundamental paradigm shift in education and academia must also occur.

## Training and Career Advancement

A key point of contention for the progression of translational research is the length of time and resources required to carry out these, often, multiyear projects. A truly translational study takes many steps during the preclinical stage before even making it to the clinical phase of study. How are those researchers undertaking translational efforts to be evaluated and supported? Translational study-support programs like those at MIT’s Dashpande Center for Technological Innovation, University of Southern California’s Stevens Center for Innovation, or Houston Methodist Hospital Research Institute’s Translational Research Initiatives have evolved to support this work, understanding that translational work may not generate traditional career elevating metrics. Publication in high impact journal and NIH funding are often sacrificed for intellectual property and patents, without which, translation would never be achieved. The customary anti-entrepreneurial world of academia will need a culture shift if they are to embrace this new breed of researcher focused on solving clinical problems rather than asking more questions about them. This will involve rethinking current criteria for promotion, such as high impact publications, grants, and invited lectures.

While regulatory issues and capital generation remain hurdles to translational success, a new pipeline of hybrid researchers is needed to cultivate the translational landscape. As pressure to generate revenue overcomes many clinicians, and scientists find it ever more difficult to obtain NIH and equivalent funding, clinician-scientists and applied-scientists must be trained to undertake the challenges of navigating an already treacherous path. With this must come administrative understanding that translational projects take longer and are born out of large multi-institutional and multidisciplinary teams where a researchers contributions may not be adequately represented. Along this continuum from the bedside to bench to bedside live the applied-scientist and the clinician-scientist. There needs to be more crossover and integration between the “two cultures” Snow described over 50 years ago. Many times thought to have an identity crisis, emphasis on translational research by the NIH and large academic institutions has empowered these individuals and highlighted their need going forward. Multidisciplinary institutions are primed to carry this agenda to fruition.

Training of this new breed of researchers not only requires support and understanding but an infrastructure capable of providing a favorable and productive environment for success. Traditional training schemes continue to promote silos in both the basic scientific and clinical realms. These walls must be broken down to create the next wave of translational researchers. Already, translational research programs in the basic sciences are evolving out of the traditional schemes. From the clinical standpoint, more focus and institutional support must be given to attract physicians to engage in research previously thought to have no place in clinical work. The Mayo Clinic Graduate School Clinical and Translational Research Program and Brown’s Masters in Clinical and Translational Research are two examples of multidisciplinary institutions incorporating translational research into their curriculums. So too has the American Board of Surgery embraced these initiative by offering flexible pathways capable of turning out a new generation of surgeon-scientists poised to engage in the translational initiatives of the institutions they serve. Alternatively, there needs to be tracks where scientists engage the clinic to truly understand the disease they are trying to impact. For transitional research to succeed and translational researchers to emerge from the traditional systems of education, there must be a paradigm shift in training and career development that lays the foundation for funding and commercialization of breakthroughs. Breakthroughs, though, are plagued by a sequential set of hurdles and valleys that must be navigated in order to bring a novel scientific breakthrough to the community.

## Death Valleys of Translational Progress

**Figure 2:**
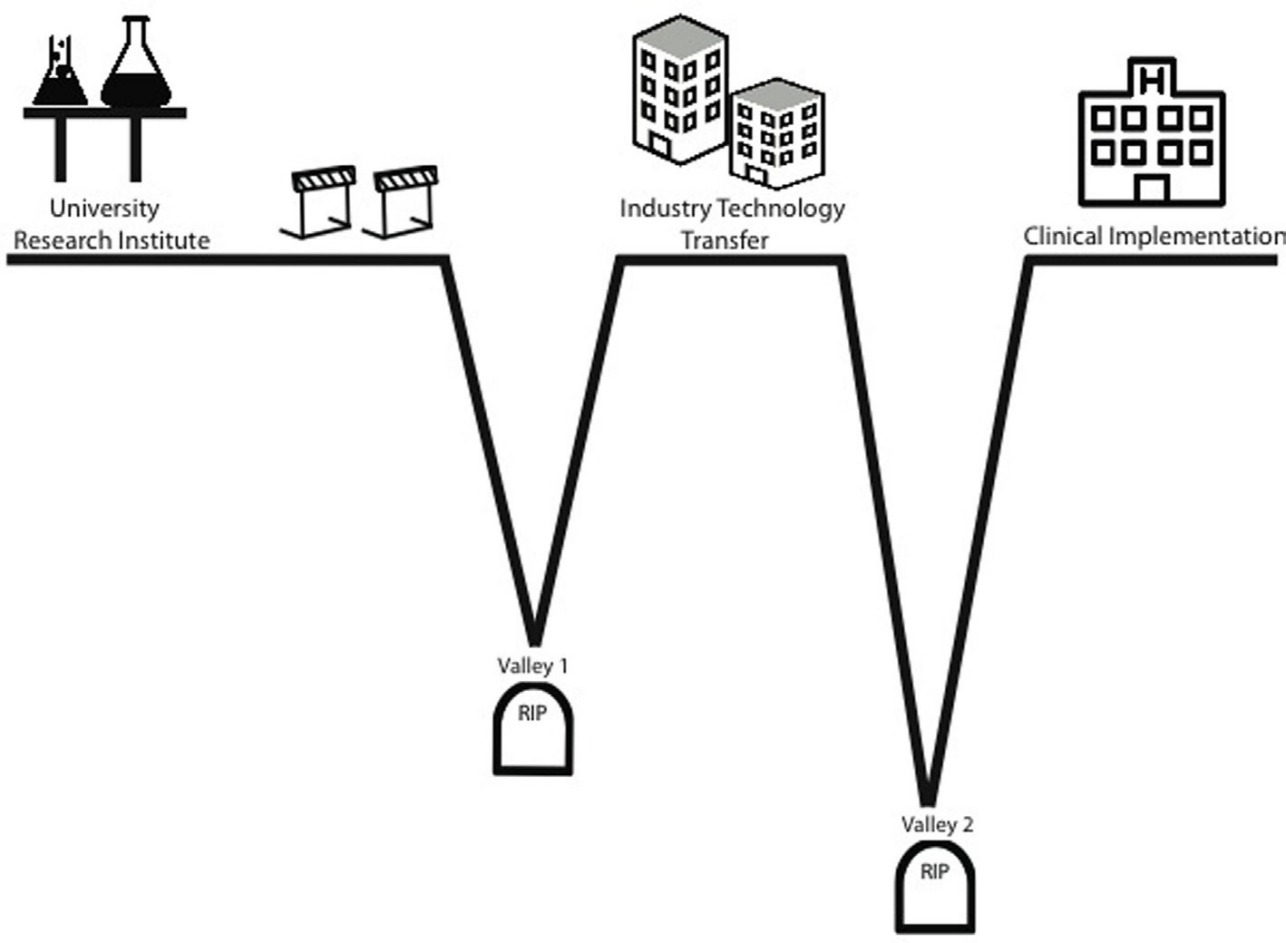
**Valleys of death**. The “valleys of death” concept is used to describe situations where technology failed to reach clinical implementation. Termination of studies in Valley 1 when a technology has shown efficacy, yet is unable to obtain financing to take it to commercialization and human trials. Termination of studies in Valley 2 is due to a rapid decline of funding during the costly human trial phase and occurs during the stage in-licensed technology becomes an actual revenue-generating product.

Novel breakthroughs capable of funding are exciting when they occur. They stir up enthusiasm, bring attention to institutions, and generate donor revenue (Heinze et al., 2009). For the relatively few advancements developed by researchers at institutions where one can out-license the intellectual property, two “valleys of death” await the inventors (Figure 2) (Roberts et al., 2012). The first valley of death is the time between the initial discovery and the actual out-licensing of the invention. The reason why inventions die in this valley is the same reason why many never occur in the first place, a lack of resources. While the researcher may have done due diligence to prove the *in vitro* and *in vivo* efficacy of the invention, the institution within which they work does not have the resources to efficiently perform the duties needed to protect the discovery. Conversely, often the research while in possession of a potential blockbuster product may not have the resources to execute the comprehensive *in vitro* and *in vivo* preclinical studies required to fully demonstrate efficacy (Meslin et al., 2013).

From the standpoint of industry, this period is seen in companies where initial trials drains funds to the point where prospect drugs or device never made it to market because they simply ran out of money to finish the trials (Murphy and Edwards, 2003). The second valley occurs, here, during the in-licensing and trials phase. There are few funding mechanisms specifically geared to support product-related translational efforts. US Small Business Innovation Research/Small Business Technology Transfer program is designed to support these efforts but is intended for small for-profit entities where the investigator is in the company not in an institution. Thus, most funding for novel biomedical tools originating at institutions must come from the private sector (Murphy and Edwards, 2003). Holders in private investment funds expect a return on their investment. They understand the risky nature of biomedical enterprise and thus, require substantial return to compensate for risk. This, again, leads to loss of funding when clinical outcomes do not reflect preclinical efficacy. Thus, we are again left with inconsistent support to move a potential life-saving therapy into clinical implementation.

## Catalysts for Translation

Bioengineering has entered a transformational era where unprecedented breakthroughs and advances hold the promise of revolutionizing health-care practice and delivery. At the threshold stands the translational scientist poised to play the critical role in the bridging the gap of cultures and yet, as mentioned above, hurdles and “valleys of death” plague advancement. The NIH has played an integral role in facilitating these collaborations as early as the 1980s. Through the Bayh–Dole Act and the Stevenson–Wydler Act, they strengthened the incentives for academic and government institutions to engage with industry in research collaborations and partnerships (Schacht, 2000; Mowery et al., 2001). Through this legislation, public sector research institutions were allowed to own the intellectual property they generated using federal funding and license it for commercialization. Through this, institutions have been able to make effective partnerships with the private sector, leading to such advancements as the creation and patenting of biologic drug candidates and small molecules (Collins and Varmus, 2015).

As drug and device companies continue to cut research and development funding and the NIH budget continues to decline, the new hybrid of public–private entity relationships may be the backbone of translational research. Programs, such as the Defense Advanced Research Projects Agency’s (DARPA) Armed Forces Institute of Regenerative Medicine (AFIRM), focus on translational initiative that will serve military clinical needs. With a focus on combat trauma and regenerative medicine solutions, DARPA has invested in research with a clear path from discovery to commercialization. The multimillion-dollar consortium collaborations born out of AFIRM are only considered for funding when a commercial entity willing to undertake the commercial manufacturing is partnered with the researchers. Launched in 2014, an emerging NIH-driven initiative is the accelerating medicine partnership (AMP). While not currently focused on materials development, the partnership, which is composed of the NIH, 10 biopharmaceutical companies, and several non-profit organizations, has raised over $180 million. A unique and groundbreaking element of this public–private partnership is the agreement to make all data analyses made during the collaboration publicly available for use by the broader health-care community. Under the current fiscal limitations of both sectors this relationship poises itself to be mutually beneficial for not only those involved but those in the medical community. As part of the greater medical knowledge explosion, collaborations such as these will rapidly accelerate the available data, leading to a greater exponential growth of scientific knowledge.

## From the Bedside to the Bench and Back: Rethinking Translation

As we rethink the dogma of scientific research around the world, we must embrace the new paradigm of translational research and realize that the journey begins and ends at the bedside. As a health-care community, we must approach our practice honestly to admit and identify shortcomings of our care so that we may turn to our applied-scientist or clinician-scientist colleagues for solutions to these shortcomings. As a scientific community, we must open our labs to integrated inter-institutional, multidisciplinary collaborations where we acknowledge and reward those undertaking the brave task of developing solutions, rather than more questions. As administrators, teachers, and mentors, we must continue to invest in the new wave of researchers who may not fit into the traditional paradigm of academic advancement and support the long road of work they have before them. As government agencies, we must continue to build partnerships with the private sector to harness the strengths of all parties. Lastly, as a whole medical community, we must embrace each other with a solitary unifying goal: to act on behalf of our patients and provide solutions to their unsolved clinical needs.

## Author Contributions

The sole author, JF-M, wrote, edited, conceived the paper, and created the figures.

## Conflict of Interest Statement

The author declares that the research was conducted in the absence of any commercial or financial relationships that could be construed as a potential conflict of interest.
